# Supplementary material for: Modeling stochasticity and variability in gene regulatory networks
Source: EURASIP J Bioinform Syst Biol. 2012 Jun 6;2012(1):5. doi: 10.1186/1687-4153-2012-5 (PMC3419641; doi:10.1186/1687-4153-2012-5)
Supplement: Additional file 1 — contains Supporting Material. [file 1687-4153-2012-5-S1.PDF]

Supporting Material for Modeling Stochasticity and Variability in Gene Regulatory Networks

David Murrugarra[a,b], Alan Veliz-Cuba[c], Boris Aguilar[d], Seda Arat[a,b], Reinhard Laubenbacher[a,b]

March 25, 2012

[a]Department of Mathematics, Virginia Tech,
Blacksburg, VA 24061-0123, USA
[b]Virginia Bioinformatics Institute, Virginia Tech,
Blacksburg, VA 24061-0477, USA
[c]Department of Mathematics, University of Nebraska Lincoln,
Lincoln, NE 68588, USA
[d]Department of Computer Science, Virginia Tech,
Blacksburg, VA 24061-0123, USA

1 Stochastic Discrete Dynamical Systems: (SDDS)

Let $x_1, \ldots, x_n$ be variables which can take values in finite sets $X_1, \ldots, X_n$, respectively. Let $X = X_1 \times \cdots \times X_n$ be the Cartesian product. A *stochastic discrete dynamical system* in the variables $x_1, \ldots, x_n$ is a collection of n triplets

$$F = \{f_i, p_i^{\uparrow}, p_i^{\downarrow}\}_{i=1}^n$$

Let $m_1, \ldots, m_n$ be the number of elements of finite sets $X_1, \ldots, X_n$, respectively. Let $m = m_1 \times \cdots \times m_n$. The state space of $F = \{f_i, p_i^{\uparrow}, p_i^{\downarrow}\}_{i=1}^n$ over X consists of m states. The network may transition from one state to other possible states. Algorithm 1.1 describes how to calculate a next state starting from an initialization. The probability of transition from state $x = (x_1, \ldots, x_n)$ to state $y = (y_1, \ldots, y_n)$ is calculated using Algorithm 1.2.

1.1 SDDS includes trajectories from the synchronous and asynchronous schemes

If $F = \{f_i, p_i^{\uparrow}, p_i^{\downarrow}\}_{i=1}^n$ a stochastic discrete dynamical system, then F has the same steady states as the deterministic system $G = \{f_i\}_{i=1}^n$. This follows

from the fact that

$$prob(x \to x) = 1 \iff prob(x_i \to x_i) = 1 \, \forall i \iff x_i = f_i(x) \iff f(x) = x.$$

Note that $prob(x \to y) = prob(x_1 \to y_1) \cdots prob(x_n \to y_n)$. Above, '$\iff$' means 'if and only if'.

It is also important to note that the dynamics of F includes all the different dynamics that can be generated from G using different update schemes. For instance, for the synchronous case, consider x, y such that $f(x) = y$, that is $f_i(x) = y_i$ for all $i = 1, \ldots, n$. There are two cases to consider:

- If $x_i = y_i = f_i(x)$, then $prob(x_i \to y_i) = 1$.
- If $x_i \neq y_i = f_i(x)$, then $prob(x_i \to y_i) = (p_i^{\uparrow}$ or $p_i^{\downarrow}) > 0$.

In any case, $prob(x_i \to y_i) > 0$. Then,

$$prob(x \to y) = \text{product(positive numbers)} > 0.$$

Then $x \to y$ is an edge in the state space of the SDDS. Note that if $p_i^{\uparrow} = p_i^{\downarrow} = 1$ for all $i = 1, \ldots, n$, then F is a synchronous deterministic system.

For the asynchronous case: assume all propensities are in $(0, 1)$. Consider x, y such that $x_i = y_i$ for all $i \neq k$ and $f_k(x) = y_k \neq x_k$. For $i \neq k$: two cases:

- if $x_i = y_i = f_i(x)$, then $prob(x_i \to y_i) = 1$.
- if $x_i = y_i \neq f_i(x)$, then $prob(x_i \to y_i) = (1 - p_i^{\uparrow}$ or $1 - p_i^{\downarrow}) > 0$.

In any case $prob((x_i \to y_i) > 0$. For $i = k$: then $prob((x_i \to y_i) = (p_i^{\uparrow}$ or $p_i^{\downarrow}) > 0$. Then, for all $i = 1, \ldots, n$ we have $prob(x_i \to y_i) > 0$. Then,

$$prob(x \to y) = \text{product(positive numbers)} > 0.$$

Therefore $x \to y$ is an edge in the state space of the SDDS.

1.2 SDDS can be written as a PBN but needs more parameters

For $n = 2$, $X = \{0, 1\} \times \{0, 1\}$, $F = (f_1, f_2) : X \to X$, where

x_1	x_2	f_1	f_2
0	0	0	0
0	1	1	0
1	0	0	1
1	1	1	0

and

	x_1	x_2
Activation	.1	.5
Degradation	.2	.9

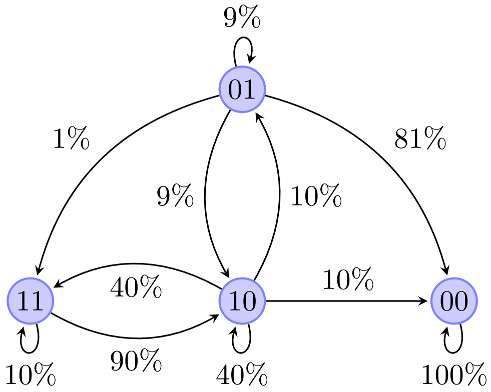

Now, suppose that there is a PBN that has the same state space. Let us focus on the Boolean functions for the first variable, which we denote $F_1 = \{h_1, h_2, \ldots, h_r\}$. Notice that for the first variable we have the following transitions:

$$Pr(00 \to 0) = 1.$$

$$Pr(01 \to 0) = .9 = 1 - p_1^{\uparrow}$$

$$Pr(01 \to 1) = .1 = p_1^{\uparrow}$$

$$Pr(10 \to 0) = .2 = p_1^{\downarrow}$$

$$Pr(10 \to 1) = .8 = 1 - p_1^{\downarrow}$$

$$Pr(11 \to 1) = 1$$

From these equalities it follows that the number of Boolean functions for the first variable is 4 (all other Boolean functions would have probability 0), namely:

x_1	x_2	h_1	h_2	h_3	h_4
0	0	0	0	0	0
0	1	0	0	1	1
1	0	0	1	0	1
1	1	1	1	1	1

Now, denote with $p1, p2, p3, p4$ the probabilities corresponding to h_1, h_2, h_3, h_4, resp. Then, we have the following equations:

$$.1 = p3 + p4,$$

$$.8 = p2 + p4,$$

and

$$p1 + p2 + p3 + p4 = 1.$$

It follows that

$$p2 = .9 - p1, p3 = .2 - p1, p4 = p1 - .1$$

If we want to keep the number of functions in the PBN as small as possible, we can consider $p3 = 0$ or $p4 = 0$ (but not both). Hence, the PBN needs at least 3 Boolean functions for the first variable.

Now, suppose that a PBN has the same state space and let us focus on the Boolean functions for the second variable, which we denote $F_2 = \{g1, g2, \ldots, gm\}$. Notice that for the second variable we have the following transitions:

$$prob(00- > 0) = 1$$

$$prob(01- > 1) = .1 = 1 - p_2^{\downarrow}$$

$$prob(01- > 0) = .9 = p_2^{\downarrow}$$

$$prob(10- > 1) = .5 = p_2^{\uparrow}$$

$$prob(10- > 0) = .5 = 1 - p_2^{\uparrow}$$

$$prob(11- > 1) = .1 = 1 - p_2^{\downarrow}$$

$$prob(11- > 0) = .9 = p_2^{\downarrow}$$

From these equalities it follows that the number of Boolean functions for the first variable is 3 (all other Boolean functions would have probability 0), namely:

x_1	x_2	g_1	g_2	g_3
0	0	0	0	0
0	1	0	0	1
1	0	0	1	1
1	1	1	1	0

$$p2 = .1,$$

$$p2 + p3 = .5,$$

$$p2 = .1,$$

$$p1 + p2 + p3 = 1,$$

Then,

$$p1 = .5, p2 = .1, p3 = .4$$

This shows that the PBN needs at least 3 Boolean functions for the second variable. Therefore, the PBN needs a total of at least 6 Boolean functions and 6 parameters. Notice that the SDDS has only 2 functions and 4 parameters.

In general,
SDDS: n func, 2n pars.
PBN: 3n func, 3n pars.

1.3 Pseudocodes

Algorithm 1.1 describes how to calculate a next state starting from an initialization. The probability of transition from state $x = (x_1, \ldots, x_n)$ to state $y = (y_1, \ldots, y_n)$ is calculated using Algorithm 1.2.

2 Regulation in the p53-Mdm2 network

The p53-Mdm2 network is one of the most widely studied gene regulatory networks. W. Abou-Jaude, D. Ouattara, M. Kauffman [1] proposed a logical four-variable model to describe the dynamics of the tumor suppressor protein p53 and its negative regulator Mdm2 in the presence and absence of DNA damage. The wiring diagram of this model is represented in Figure 1, where P, Mc, Mn, and Dam stand for protein p53, nuclear Mdm2, cytoplasmic Mdm2, and DNA damage, respectively.

Algorithm 1.1 Next state for Stochastic Discrete Dynamical Systems

input : Initial state x_0, and a SDDS $F = \{f_i, p_i^{\uparrow}, p_i^{\downarrow}\}_{i=1}^{n}$.
output: y = one of the next states of x.

Let $z = F(x_0) = (f_1(x_0), \ldots, f_n(x_0))$.
for $i = 1$ **to** n **do**
    Let r *be a random number in [0,1].*
    if $x_i < z_i$ **then**
        if $r < p_i^{\uparrow}$ **then**
            $y_i = z_i$ with probability $p_i^{\uparrow}$
        else
            $y_i = x_i$ with probability $1 - p_i^{\uparrow}$
        end
    else if $x_i > z_i$ **then**
        if $r < p_i^{\downarrow}$ **then**
            $y_i = z_i$ with probability $p_i^{\downarrow}$
        else
            $y_i = x_i$ with probability $1 - p_i^{\downarrow}$
        end
    else
        $y_i = x_i$ with probability 100%
    end
end

Algorithm 1.2 Transition probability between two states for SDDS.

input : Two states x, y, and a SDDS $F = \{f_i, p_i^{\uparrow}, p_i^{\downarrow}\}_{i=1}^{n}$.
output: $P_{x,y}$ = The probability of transitioning from x to y.

Let $z = F(x) = (f_1(x), \ldots, f_n(x))$.
$P_{x,y}$ *= 1.*
for $i = 1$ **to** n **do**
    Let $c = 0$.
    if $x_i < z_i$ **then**
        if $y_i = z_i$ **then**
            $c = p_i^{\uparrow}$
        end
        if $y_i = x_i$ **then**
            $c = 1 - p_i^{\uparrow}$
        end
    else if $x_i > z_i$ **then**
        if $y_i = z_i$ **then**
            $c = p_i^{\downarrow}$
        end
        if $y_i = x_i$ **then**
            $c = 1 - p_i^{\downarrow}$
        end
    else
        if $y_i = x_i$ **then**
            $c = 1$
        end
    end
    $P_{x,y} = P_{x,y} * c$
end

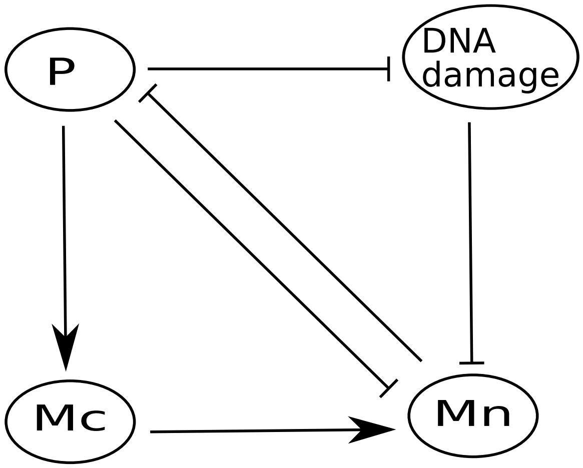

Figure 1: Four-variable model for the p53-Mdm2 regulatory network. P, Mc, and Mn stand for protein p53, nuclear Mdm2, and cytoplasmic Mdm2 respectively.

Table 1: Truth table for P

Mn P	P
0 0	1
0 1	2
0 2	2
1 0	0
1 1	0
1 2	1

The state space for this model is specified by $[0,2] \times [0,1] \times [0,1] \times [0,1]$, that is, except for the first variable P that has three levels $\{0,1,2\}$, all the other variables are Boolean.

As shown in Figure 1, Mn acts negatively on P. The update rule for P, f_P, is specified by the truth table given at Table 1

The update rule for Mc, f_{Mc}, which is specified by the truth table given at Table 2.

The update rule for Mn, f_{Mn}, which is specified by the truth table given at Table 3.

Finally, the update rule for DNA-damage, f_{Dam}, which is specified by the truth table given at Table 4.

Table 2: Truth table for Mc

P	Mc
0	0
1	0
2	1

Table 3: Truth table for Mn

P Mc Dam	Mn
0 0 0	1
0 0 1	0
0 1 0	1
0 1 1	1
1 0 0	0
1 0 1	0
1 1 0	1
1 1 1	1
2 0 0	0
2 0 1	0
2 1 0	1
2 1 1	1

Table 4: Truth tables for *DNA-damage*

P Dam	Dam
0 0	0
0 1	1
1 0	0
1 1	1
2 0	0
2 1	0

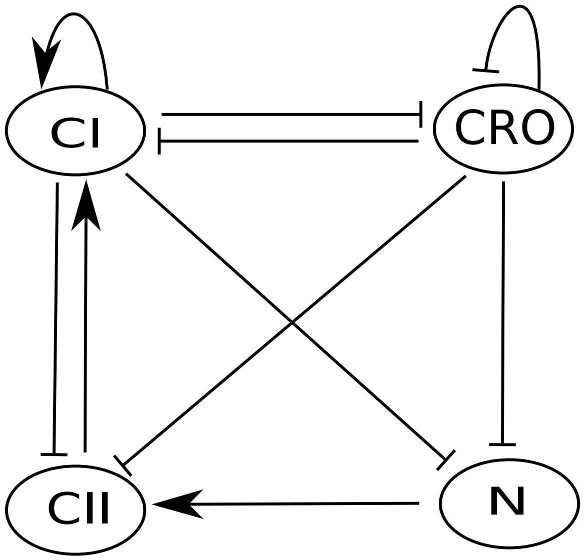

Figure 2: Four-variable model for the lambda phage regulatory network.

3 Lambda phage infection of bacteria

Thieffry and Thomas [2] built a multi-state logical model for the core lambda phage regulatory network. This model encompasses the roles of the regulatory genes *CI*, *Cro*, *CII*, and *N*. See Figure 2 .

The state space for this model is specified by $[0,2] \times [0,3] \times [0,1] \times [0,1]$, that is, the first variable has three levels $\{0,1,2\}$, the second variable has four levels $\{0,1,2,3\}$, and the third and fourth variables are still Boolean.

The update rule for CI, f_{CI}, has inputs CRO and CII which is specified by the truth table given at Table 5

The update rule for CRO, f_{CRO}, which is specified by the truth table given at Table 6.

The update rule for CII, f_{CII}, which is specified by the truth table given at Table 7.

Finally, the update rule for N, f_N, which is specified by the truth table given at Table 8.

Table 5: Truth table for CI

CRO CII	CI
0 0	2
0 1	2
1 0	0
1 1	2
2 0	0
2 1	2
3 0	0
3 1	2

Table 6: Truth table for CRO

CI CRO	CRO
0 0	3
0 1	3
0 2	3
0 3	2
1 0	3
1 1	3
1 2	3
1 3	2
2 0	0
2 1	0
2 2	0
2 3	0

Table 7: Truth table for CII

CI CRO N	CII
0 0 0	0
0 0 1	1
0 1 0	0
0 1 1	1
0 2 0	0
0 2 1	1
0 3 0	0
0 3 1	0
1 0 0	0
1 0 1	1
1 1 0	0
1 1 1	1
1 2 0	0
1 2 1	1
1 3 0	0
1 3 1	0
2 0 0	0
2 0 1	0
2 1 0	0
2 1 1	0
2 2 0	0
2 2 1	0
2 3 0	0
2 3 1	0

Table 8: Truth tables for N

CI CRO	N
0 0	1
0 1	1
0 2	0
0 3	0
1 0	0
1 1	0
1 2	0
1 3	0
2 0	0
2 1	0
2 2	0
2 3	0

4 Relating the propensity parameters with biological information

Consider the following simple degradation model from [3], page 40,

$$S \xrightarrow{k} \emptyset$$

The propensity function (in the Gillespie context) is $a(x) = kx$ and the state vector change is $\nu = -1$. The expected value of the solution of this stochastic model is given by $X(t) = x_0 \exp(-kt)$, where $X(t)$ represent the state of the system at time t.

Let us discretize the number of states even further, into two states 0 and 1.

$$\tilde{X}(t) = \begin{cases} 0 & X(t) < m \\ 1 & X(t) \geqslant m \end{cases}$$

where m is a fraction of the initial number of molecules, i.e. $m = \frac{x_0}{\eta}$. Now, from the expected value estimate we have

$$m = \frac{x_0}{\eta} = x_0 \exp(-k\tilde{t}) \text{ for some } \tilde{t}.$$

then

$$\ln(\frac{1}{\eta}) = -k\tilde{t}.$$

then

$$\frac{\ln(\eta)}{k} = \tilde{t}.$$

The probability distribution for the time to go from 1 to 0 in SDDS is a geometric distribution [4] (Pg. 65) with expected value $1/p_1^{\downarrow}$, i.e. the average 'waiting time' to go from 1 to 0 in SDDS is $1/p_1^{\downarrow}$. Therefore

$$\frac{\ln(\eta)}{k} \approx \delta \frac{1}{p_1^{\downarrow}}.$$

Then

$$p_1^{\downarrow} \approx \frac{\delta}{\ln(\eta)} k$$

Therefore

$$p_1^{\downarrow} \approx ck.$$

where $c = \frac{\delta}{\ln(\eta)}$.

In Figure 3 we compare SDDS simulations with Gillespie simulations. In order to fit both plots in a single figure, we have normalized the number of molecules so that it goes from 0 to 1. Number of simulations for SDDS is 1000 and number of steps for each simulation was 6. The number of molecules for Gillespie was normalized so that it goes from 0 to 1. The scale parameter δ for SDDS was set equal to 20, i.e., 1 second in the scale of Gillespie is equivalent to 20 time steps in the scale of SDDS. Thus

$$c = \frac{\delta}{\ln(\eta)} = \frac{20}{\ln(5)} = 12.4267$$

Therefore

$$p_1^{\downarrow} \approx (12.4267)(0.05) = 0.6213.$$

References

[1] W. Abou-Jaoudé, D. Ouattara, and M. Kaufman. (2009) From structure to dynamics: Frequency tuning in the p53-mdm2 network: I. logical approach. *Journal of Theoretical Biology*, 258(4):561 – 577.

[2] Thieffry, D., Thomas, R. (1995) Dynamical behaviour of biological regulatory networks–II. Immunity control in bacteriophage lambda. *Bull. Math. Biol.* 57, 277-295.

[3] D. Gillespie. (2007) Stochastic simulation of chemical kinetics. *Annu. Rev. Phys. Chem.* 58:35-55.

[4] Darren J. Wilkinson. (2006) Stochastic Modelling for Systems Biology *Chapman and Hall/CRC.*

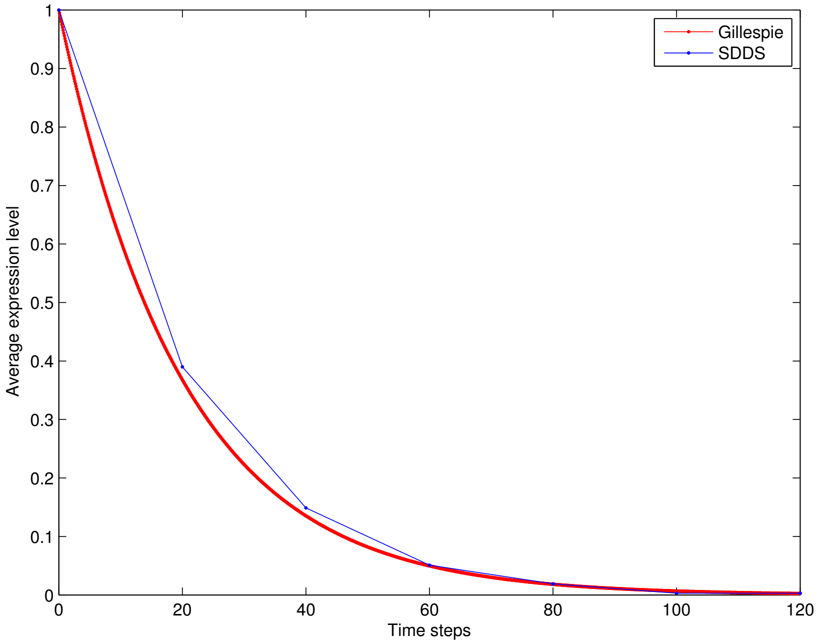

Figure 3: SDDS vs Gillespie. Number of molecules for Gillespie is 1000 and degradation rate $k = 0.05$. Number of simulations for SDDS is 1000 and number of steps for each simulation was 6. The number of molecules for Gillespie was normalized so that it goes from 0 to 1. The scale parameter δ for SDDS was set equal to 20, i.e. 1 second in the scale of Gillespie is equivalent to 20 time steps in the scale of SDDS. Thus, $p_1^{\downarrow} = 0.6213$ (see text for better description).
